# Interfacial Interaction and Sintering Mechanism of Cellulose/Polylactic Acid Composites in Selective Laser Sintering: A Molecular Dynamics Simulation

**DOI:** 10.3390/polym18151859

**Published:** 2026-07-29

**Authors:** Yibing Tian, Xirui Yang, Haoyu Zhang, Runan Gong, Li Zou, Hong Zhang, Yanling Guo

**Affiliations:** 1 College of Mechanical and Electronic Engineering, Tarim University, Alar 843300, China; 10757242232@stumail.taru.edu.cn (Y.T.); 10757231137@stumail.taru.edu.cn (X.Y.); 10757251167@stumail.taru.edu.cn (R.G.); 2 Modern Agricultural Engineering Key Laboratory at Universities of Education Department of Xinjiang Uygur Autonomous Region, Alar 843300, China; 3 Xinjiang Production and Construction Corps Key Laboratory of Utilization and Equipment of Special Agricultural and Forestry Products in Southern Xinjiang, Alar 843300, China; 4 College of Mechanical and Electrical Engineering, Northeast Forestry University, Harbin 150040, China; zhy119803@163.com; 5 College of Materials Science and Engineering, Northeast Forestry University, Harbin 150040, China; zouli@nefu.edu.cn

**Keywords:** molecular dynamics simulation, polylactic acid, cellulose, selective laser sintering, interfacial interaction

## Abstract

Polylactic acid (PLA) is a biodegradable polymer suitable for selective laser sintering, yet the atomic-level sintering mechanisms of plant-fiber-reinforced PLA remain largely unclear. Here, all-atom molecular dynamics simulations are used to investigate the sintering behavior and interfacial interactions of cotton stalk cellulose/PLA nanocomposites with cellulose contents of 0–20 wt%. The nanoparticle coalescence proceeds through three sequential stages: initial van der Waals-driven contact, thermally activated neck formation, and isothermal interfacial densification. Cellulose content exerts a non-monotonic effect on interfacial bonding. At 15 wt% cellulose, an optimal, continuous hydrogen-bond network forms between cellulose hydroxyls and PLA carbonyls, maximizing interfacial adhesion while simultaneously suppressing unfavorable dipole stacking among PLA chains. At this critical loading, cellulose acts as a rigid scaffold that directs PLA chain extension and directional migration across the sintering neck, as evidenced by atomic displacement and radius-of-gyration analyses. In contrast, excess cellulose (20 wt%) induces self-aggregation, which disrupts interfacial continuity and hinders long-range chain diffusion. These results identify 15 wt% as the optimal cellulose content for achieving enhanced interfacial bonding and chain mobility, providing atomistic criteria for the formulation design of cellulose/PLA composite powders for selective laser sintering processing.

## 1. Introduction

Polylactic acid (PLA) is one of the most promising biodegradable alternatives to conventional petroleum-based polymers, owing to its good mechanical strength, renewability, and biocompatibility [1,2,3]. However, its inherent brittleness, poor impact resistance, and relatively slow degradation rate restrict broader applications. Incorporation of plant fibers—such as flax, bamboo, and cotton stalk—into PLA matrices has been demonstrated to enhance the stiffness, strength, and thermal stability of the resulting composites [4,5,6,7,8], as the dispersed fibers form a reinforcing network that improves mechanical performance and modulates degradation behavior [9]. PLA compounded with plant fibers exhibits predictable, reproducible, and tunable characteristics, and its 3D-printed products have found diverse applications [10,11,12,13,14].

Selective laser sintering (SLS), a powder bed fusion additive manufacturing technique, offers distinct advantages for fabricating polymer components with complex geometries, including high forming precision, excellent material utilization, and solvent-free processing [15,16,17]. During SLS, polymer particles undergo laser heating, melting, coalescence, and densification, processes governed by particle size, thermal history, chain mobility, and interfacial interactions [18,19]. A mechanistic understanding of these phenomena is therefore essential for optimizing SLS processing of polymer composites [20].

Plant fiber/PLA composites have attracted growing interest because of their complete biodegradability and effective mechanical reinforcement [21,22,23]. Nevertheless, experimental studies alone cannot resolve the atomic-scale events during sintering, such as chain diffusion, interfacial hydrogen-bonding dynamics, and the evolution of molecular conformations. Molecular dynamics (MD) simulations provide a powerful means to bridge this gap. Recent MD studies have investigated PLA nanoparticle interactions [24], bamboo fiber/PBS composites [25], and mechanical properties of 3D-printed PLA composites [26], as well as cellulose combustion mechanisms [27]. These works, however, have largely focused on static property characterization or distinct chemical processes, leaving the dynamic coalescence behavior, chain migration patterns, and interfacial evolution of cellulose/PLA composites during SLS largely unexplored [28,29,30]. In particular, three interrelated questions remain inadequately explained: (i) how cellulose content influences the formation and growth of sintering necks, (ii) how the connectivity of the interfacial hydrogen-bond network evolves with cellulose loading, and (iii) whether excessive cellulose induces self-aggregation that impedes chain diffusion and interfacial coalescence.

To address these questions, the present work employs all-atom molecular dynamics simulations to systematically investigate the sintering behavior and interfacial interactions of cotton stalk cellulose/PLA composites during the SLS process. Specifically, the kinetic stages of coalescence are first characterized to address question (i), the composition-dependent interfacial hydrogen-bond network and cohesive energy are quantified to address question (ii), and the conformational rearrangements and atomic migration pathways of PLA chains are analyzed to clarify the role of cellulose as a structural modifier, thereby addressing question (iii). By systematically correlating cellulose loading with both interfacial energetics and chain dynamics, this work intends to provide a mechanistic basis for the rational composition design of cellulose/PLA composite powders for SLS processing.

## 2. Materials and Methods

### 2.1. Force Field Selection and Validation

This study adopts a multi-level force field strategy to decouple the static structural equilibration of molecular models from the simulation of the dynamic sintering process. The construction and equilibrium relaxation of the initial amorphous models were performed on the Materials Studio platform using the COMPASS III force field, which has been systematically optimized for bonding parameters, charge distributions, and vdW parameters of organic molecules and polymers composed of light elements such as C, H, and O. This force field can accurately predict the conformations, vibrational spectra, and thermophysical properties of polymer systems [27].

The dynamic sintering simulations were carried out using the Reactive Force Field (ReaxFF) within the LAMMPS platform. ReaxFF describes chemical reaction processes based on bond order, enabling the simulation of covalent bond formation and breaking without predefining reaction pathways. The ReaxFF parameter set employed in this study has been systematically trained and validated for organic molecules containing C, H, and O, and is suitable for describing the bonding dynamics of PLA and cellulose under thermally activated conditions. For the treatment of long-range electrostatic interactions, the Ewald summation method was employed in all ReaxFF simulations. In this approach, the Coulombic interactions are decomposed into a short-range real-space component and a long-range reciprocal-space component, the latter being efficiently evaluated via the particle–particle–particle-mesh algorithm. This methodology ensures accurate description of the electrostatic interactions under the periodic boundary conditions applied in the present simulations. This force field has been validated in similar systems, such as polymer sintering [28] and cellulose pyrolysis [26].

### 2.2. Molecular Chain Modeling

**Figure 1:**
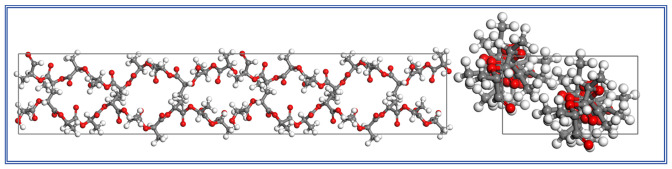
The molecular chain model of α’(δ)-PLA.

The PLA chain was modeled with a degree of polymerization of *n* = 20 using the structural formula E_1_-(C_3_H_4_O_2_)_20_-E_2_, where E_1_ and E_2_ are hydrogen-capped terminal groups. A helical conformation was constructed according to the crystallographic parameters of the α’ (δ)-PLA crystal reported in the literature [15], as illustrated in Figure 1.

**Figure 2:**
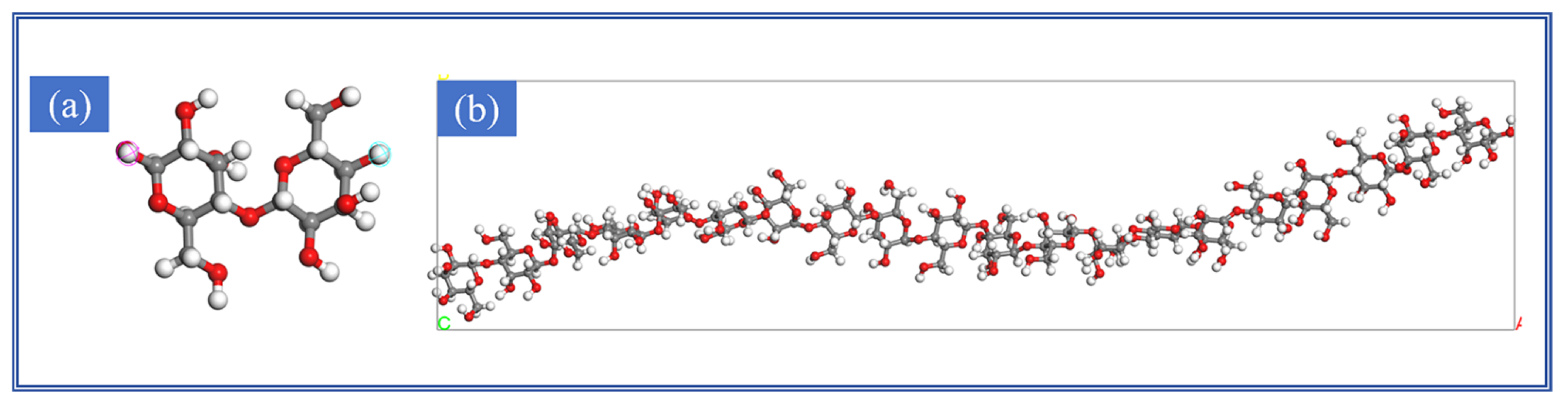
Molecular models of cellulose: (**a**) cellobiose repeating unit; (**b**) optimized cellulose chain (DP = 10) after geometric refinement.

The cellulose model was adopted from the cotton stalk cellulose structure proposed by Li Ming et al. [29], with a degree of polymerization of *n* = 10 (432 atoms per chain), as illustrated in Figure 2.

### 2.3. Construction and Equilibration of Amorphous Cell Models

The PLA molecular chains were annealed using the Anneal task in the Forcite module to obtain relaxed conformations. The annealing was performed under the NVT ensemble for 20 cycles between 300 K and 550 K with a temperature gradient of 100 K; each temperature step was simulated for 10 ps of molecular dynamics with a timestep of 1.0 fs. To avoid initial atomic overlaps and ensure the stability of the subsequent compression, an amorphous cell with an initial density of 0.15 g/cm^3^ was constructed using the Amorphous Cell module, containing 48 PLA chains, with periodic boundary conditions and an orthorhombic lattice (90 Å × 90 Å × 94.7 Å). This artificially low initial density is a standard protocol in the Amorphous Cell module for generating random chain configurations without steric clashes. The system density was then gradually increased to the true density of amorphous PLA through a stepwise compression method [30]. This target density range is well established for fully amorphous PLA from both experimental measurements and previous molecular dynamics simulations. Finally, a 50 ps relaxation was conducted under the NVT ensemble at 300 K using a Nosé-Hoover thermostat and a timestep of 1.0 fs, yielding the final equilibrated structure for subsequent property analysis.

**Figure 3:**
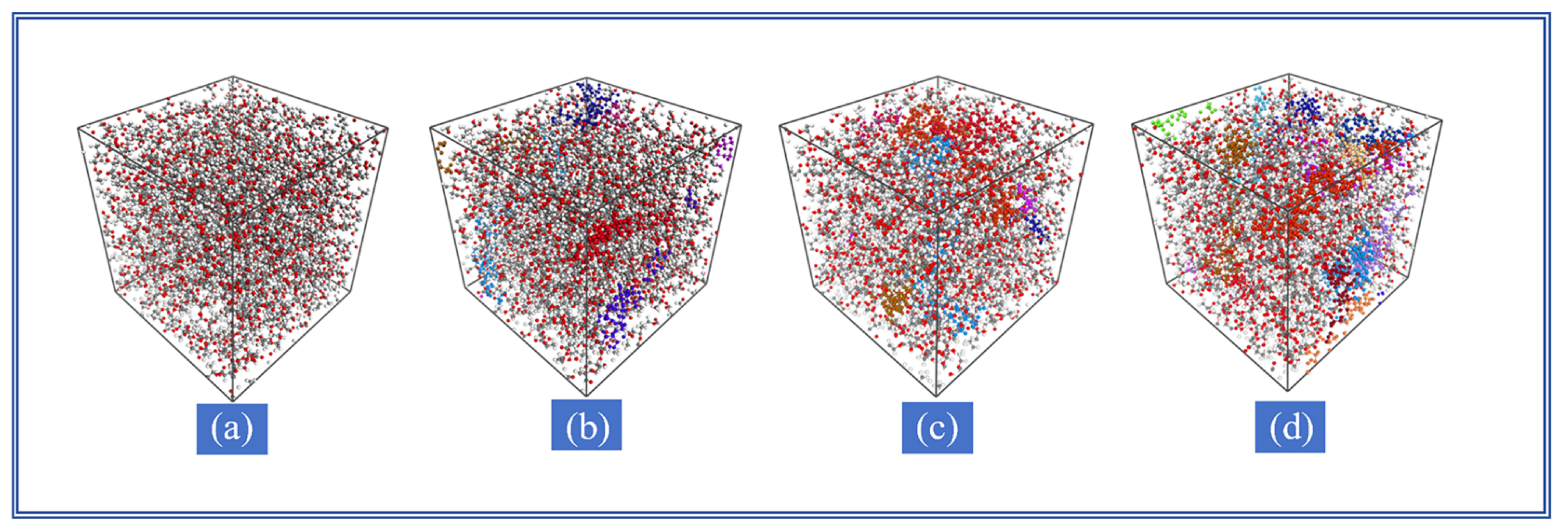
Amorphous cell models with different cellulose mass fractions: (**a**) PLA; (**b**) 10 wt% C-PLA; (**c**) 15 wt% C-PLA; (**d**) 20 wt% C-PLA. Cellulose chains are highlighted in a vivid color for clarity. The cellulose distribution evolves from isolated dispersion (10 wt%) to a percolating network (15 wt%), and further to localized aggregation (20 wt%).

Blend systems with cellulose mass fractions of 10 wt%, 15 wt%, and 20 wt% were obtained by adjusting the numbers of PLA and cellulose chains. For the pure PLA system, the amorphous cell contained 48 PLA chains (DP = 20). For the blend systems, the numbers of PLA chains were reduced to 42, 40, and 38, with 2, 3, and 4 cellulose chains (DP = 10) added to achieve cellulose mass fractions of approximately 10 wt%, 15 wt%, and 20 wt%, respectively. The resulting densities ranged from 1.225 to 1.263 g/cm^3^, with simulation box dimensions of approximately 44–45 Å in each direction. Initial configurations were generated in the Amorphous Cell module by specifying the chain numbers and target mass fractions, as illustrated in Figure 3.

### 2.4. Construction of the Double-Nanoparticle Sintering Model

**Figure 4:**
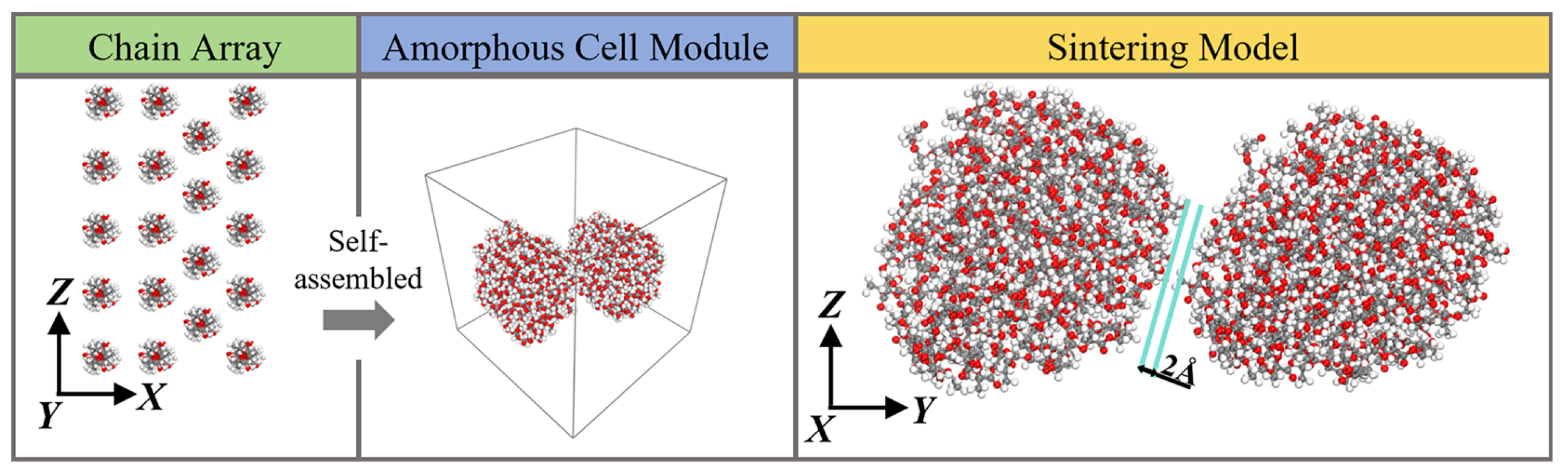
Construction procedure of the two-nanoparticle sintering model.

To investigate the dynamic sintering behavior of particles under SLS-relevant conditions, double-nanoparticle models were constructed for pure PLA and for systems with varying cellulose content. Following established methodologies, each nanoparticle was formed through the self-assembly of multiple molecular chains. For the pure PLA nanoparticle, 19 PLA chains were placed in a large simulation box and subjected to MD simulation in the NVT ensemble at 500 K using a Nosé-Hoover thermostat, allowing the chains to spontaneously aggregate into a spherical nanoparticle. The system was then cooled to 300 K and equilibrated. For the blend nanoparticles, PLA and cellulose chains were mixed in proportions corresponding to the target mass fractions. Two identical nanoparticles were subsequently placed in a cubic simulation box with dimensions of 65 Å × 65 Å × 60 Å. Periodic boundary conditions were applied in all directions to eliminate boundary effects. The initial center-of-mass distance between the two nanoparticles was set to 2 Å through iterative translation, ensuring that initial overlap was avoided while enabling early contact during the sintering process, as illustrated in Figure 4. The detailed parameters of the nanoparticle models are presented in Table 1.

### 2.5. Sintering Simulation

All sintering simulations were performed using the ReaxFF reactive force field within the LAMMPS platform. The simulation procedure consisted of three stages: (1) an initial relaxation was conducted under the NVT ensemble at 300 K for 4 ps to bring the system to thermodynamic equilibrium; (2) the system temperature was linearly ramped from 300 K to 550 K over 12 ps; (3) isothermal sintering was carried out at 550 K for 60 ps to fully capture the processes of particle melting contact, sintering neck formation, and interfacial densification. The selection of 550 K as the sintering temperature was based on the following considerations. First, this temperature exceeds the melting temperature of PLA (~450 K) by approximately 100 K, thereby imparting sufficient chain mobility and molecular diffusion capability to the PLA segments [31]. Second, thermal stability analyses of cellulose indicate that thermal degradation, primarily through dehydration reactions and glycosidic bond cleavage, begins within the range of 250–300 °C (523–573 K) and remains insignificant at approximately 550 K [32]. It is worth noting that in experimental practice, selective laser sintering of PLA is typically conducted at powder bed temperatures ranging from 160 to 220 °C (433–493 K); the simulation temperature exceeds the experimental values by approximately 60–120 K. This discrepancy originates from the overheating effects inherent in molecular dynamics simulations [33]: owing to the femtosecond-to-picosecond time scale and the ultra-fast heating rates employed, the thermal activation energy barriers for polymer segments are correspondingly elevated, necessitating a higher temperature to achieve chain mobility comparable to that observed experimentally. Therefore, the adoption of 550 K represents a reasonable compromise that accounts for the chain mobility requirements of PLA, the thermal stability limits of cellulose, and the acceleration strategy inherent in MD simulations. Throughout the simulations, all-atom trajectory data were recorded at intervals of 1 ps, providing a dynamic dataset for subsequent microscopic mechanism analysis.

## 3. Results and Discussion

### 3.1. Structural Characterization

#### 3.1.1. Atomic Potential Energy Calculation

**Figure 5:**
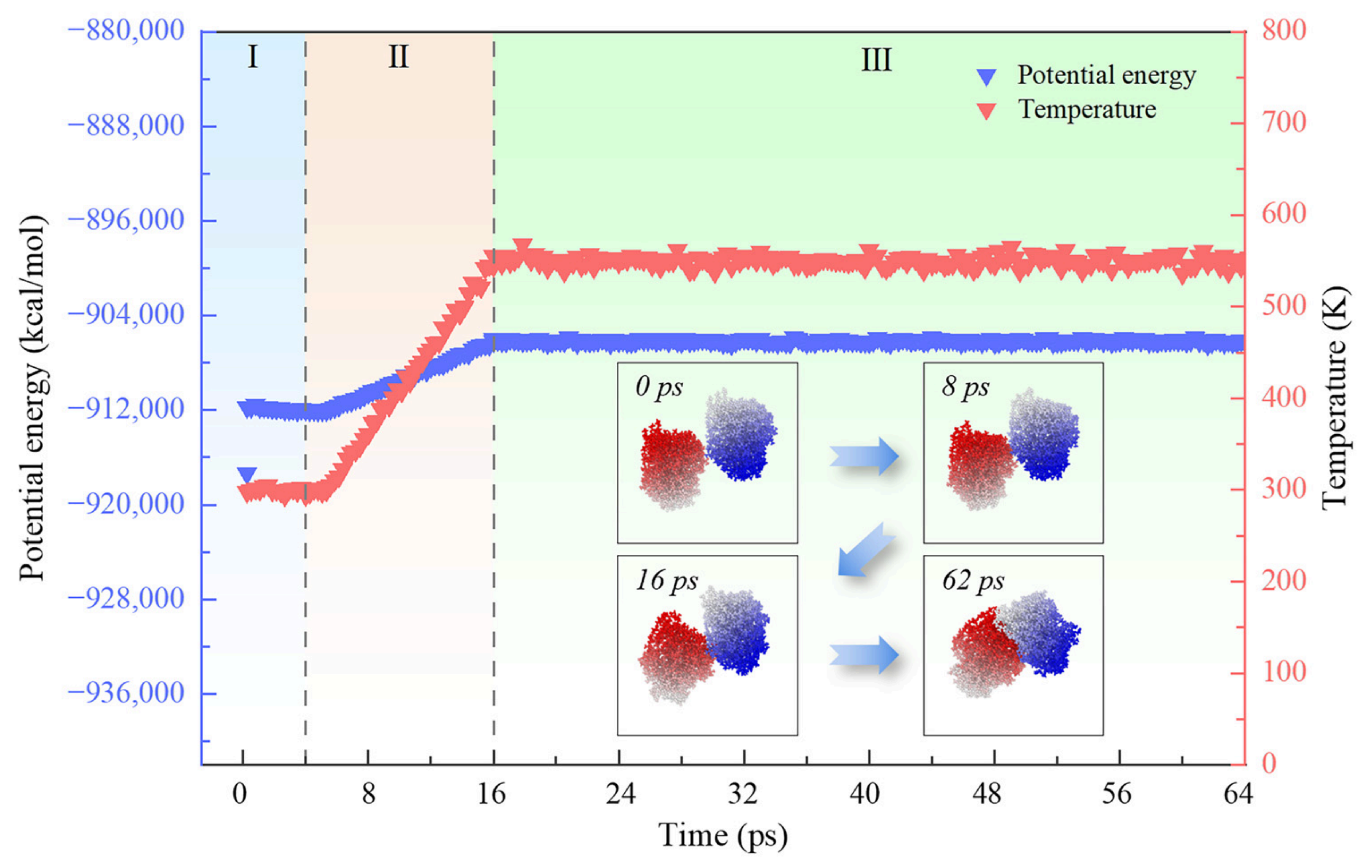
Temporal evolution of the system potential energy and temperature during the sintering process.

Figure 5 presents the evolution of the total potential energy of the entire simulation cell and the temperature as functions of time during the sintering process. The results reveal a pronounced coupling between potential energy and temperature, both of which are closely correlated with the different stages of PLA nanoparticle sintering. Concurrently, the morphological evolution of the nanoparticles is visualized through synchronized snapshots. Based on the trends in potential energy and temperature, the entire sintering process can be divided into three distinct stages: Stage I (0–4 ps), Stage II (4–16 ps), and Stage III (16–64 ps). During Stage I, the system is maintained at a constant temperature of 300 K, and vdW attractive forces between the particles drive a continuous reduction in the interparticle distance, leading to a slight decrease in system potential energy. Upon entering Stage II, the system is linearly heated to 550 K, resulting in a sharp increase in potential energy; at this stage, the kinetic energy of the nanoparticles is substantially enhanced, promoting a rapid expansion of the interatomic contact area. Ultimately, in Stage III, the two PLA nanoparticles further coalesce to form a sintered structure characterized by a central bulge and converging ends, with the system potential energy exhibiting a minor decline. These dynamic evolution characteristics are highly consistent with experimental observations [15], thereby validating the accuracy of the established MD model and the reliability of the simulation methodology.

#### 3.1.2. Radial Distribution Function

**Figure 6:**
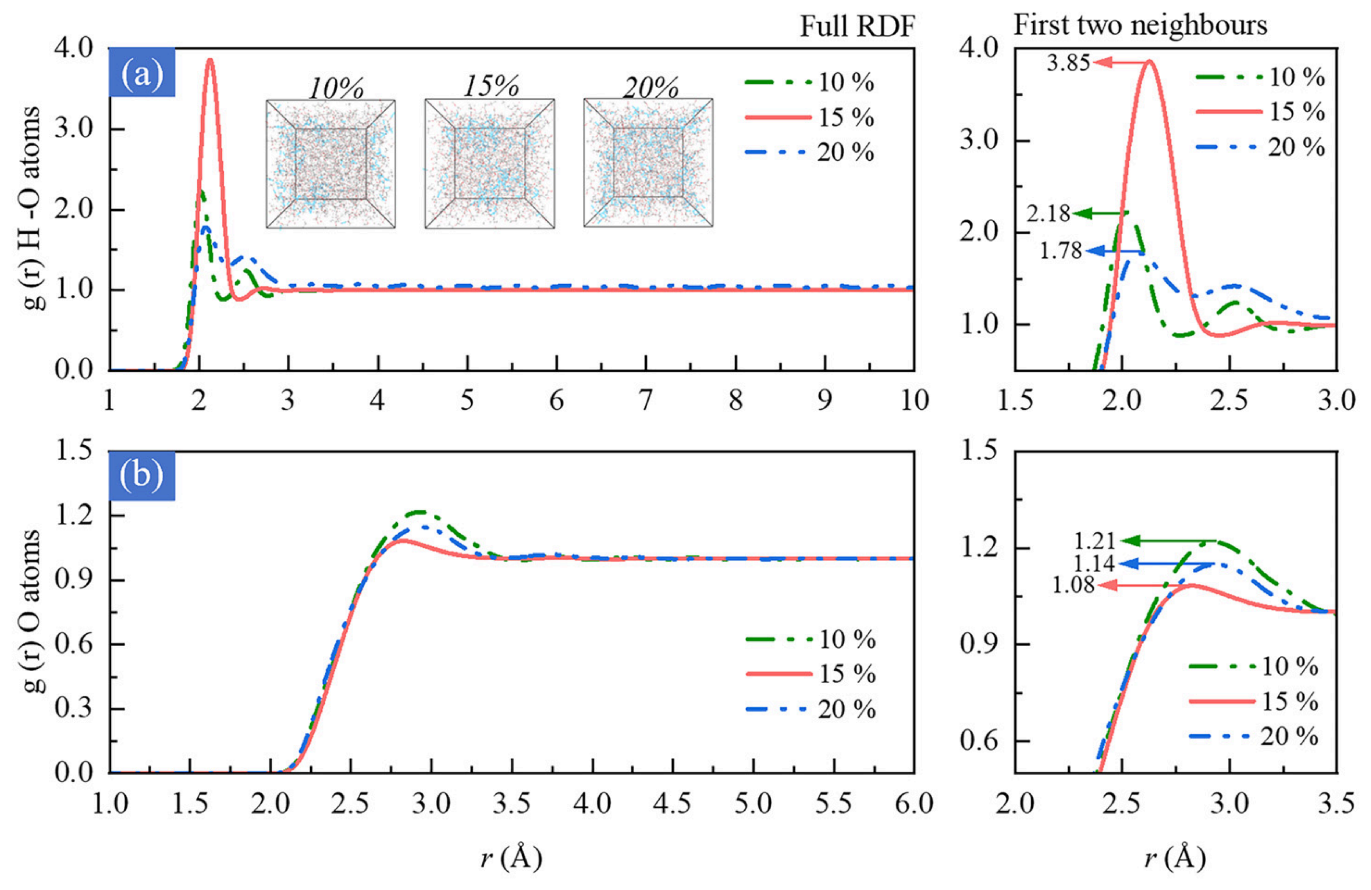
Interfacial interactions characterized by radial distribution functions: (**a**) cross-RDF g(r) between cellulose hydroxyl hydrogen (H) and PLA carbonyl oxygen (O); (**b**) auto-RDF g(r) between PLA carbonyl oxygen atoms.

The radial distribution function, g(r), describes the probability of finding an atom of a given type at a distance r from a reference atom relative to an ideal uniform distribution. A higher g(r) value indicates a greater number of atoms present within a certain distance. To quantitatively characterize the interfacial interactions between cellulose and the PLA matrix, the cross radial distribution function between the hydroxyl hydrogen atoms of cellulose and the carbonyl oxygen atoms of PLA, $g_{H\text{-}O}^{\mathrm{inter}}\left(r\right)$, was calculated, as shown in Figure 6a. All composite systems exhibit a distinct characteristic peak within the range of 1.85–2.10 Å, with a maximum at approximately 1.95 Å, indicating the presence of typical hydrogen-bonding interactions between the cellulose hydroxyl hydrogen and PLA carbonyl oxygen, corresponding to the characteristic hydrogen-bond distance commonly observed in polymer–cellulose systems. This distance range is consistent with the classic hydrogen-bond length reported for similar systems [34]. The peak intensity displays a non-monotonic evolution with increasing cellulose content: it rises from the 10 wt% value to a maximum at 15 wt% (approximately 1.8-fold higher), suggesting that the interfacial hydrogen-bond density and network connectivity are optimized at this composition, and subsequently declines to approximately half of the peak value at 20 wt%, accompanied by peak broadening and the emergence of a shoulder peak at approximately 2.7 Å. This trend is consistent across independent analyses, supporting its statistical significance. The peak broadening and the shoulder feature at 2.7 Å suggest that multiple types of atomic pair correlations coexist at higher cellulose loadings; this shoulder is tentatively assigned to the correlation between cellulose hydroxyl hydrogens and the ether oxygens of PLA backbone or to non-ideal hydrogen-bonding contacts arising from cellulose self-aggregation, which introduces structural heterogeneity at the interface. This behavior is attributed to the self-aggregation of excessive cellulose, which weakens the heterogeneous interfacial contact.

As a complementary analysis, the autocorrelation function between the carbonyl oxygen atoms of PLA,${\;g}_{O\text{-}O}\left(r\right)$, was computed, as presented in Figure 6b. The results reveal a weak, broad peak at approximately 2.8 Å for all systems, which is assigned to dipole–dipole stacking interactions between the carbonyl groups of neighboring PLA chains. in which the partial negative charges on the carbonyl oxygens give rise to local electrostatic correlations. The broad nature of this peak reflects the lack of long-range order in the amorphous PLA matrix. Notably, the intensity of this peak reaches its minimum (1.08) at 15 wt% cellulose loading, indicating that the dense cellulose network acts as a physical spacer phase that effectively suppresses ordered stacking between PLA chains and promotes the random dispersion of chain segments. When the cellulose content increases excessively to 20 wt%, self-aggregation leads to the expulsion of some PLA chains from the agglomerates, forming locally densified packing in PLA-rich regions, and consequently a slight rebound in peak height is observed.

Taken together, the cross and autocorrelation RDF analyses demonstrate that a cellulose content of 15 wt% simultaneously maximizes the interfacial hydrogen-bond network and minimizes the unfavorable dipole interactions between PLA chains, thereby achieving optimal interfacial bonding and matrix structure.

#### 3.1.3. Cohesive Energy Density

**Figure 7:**
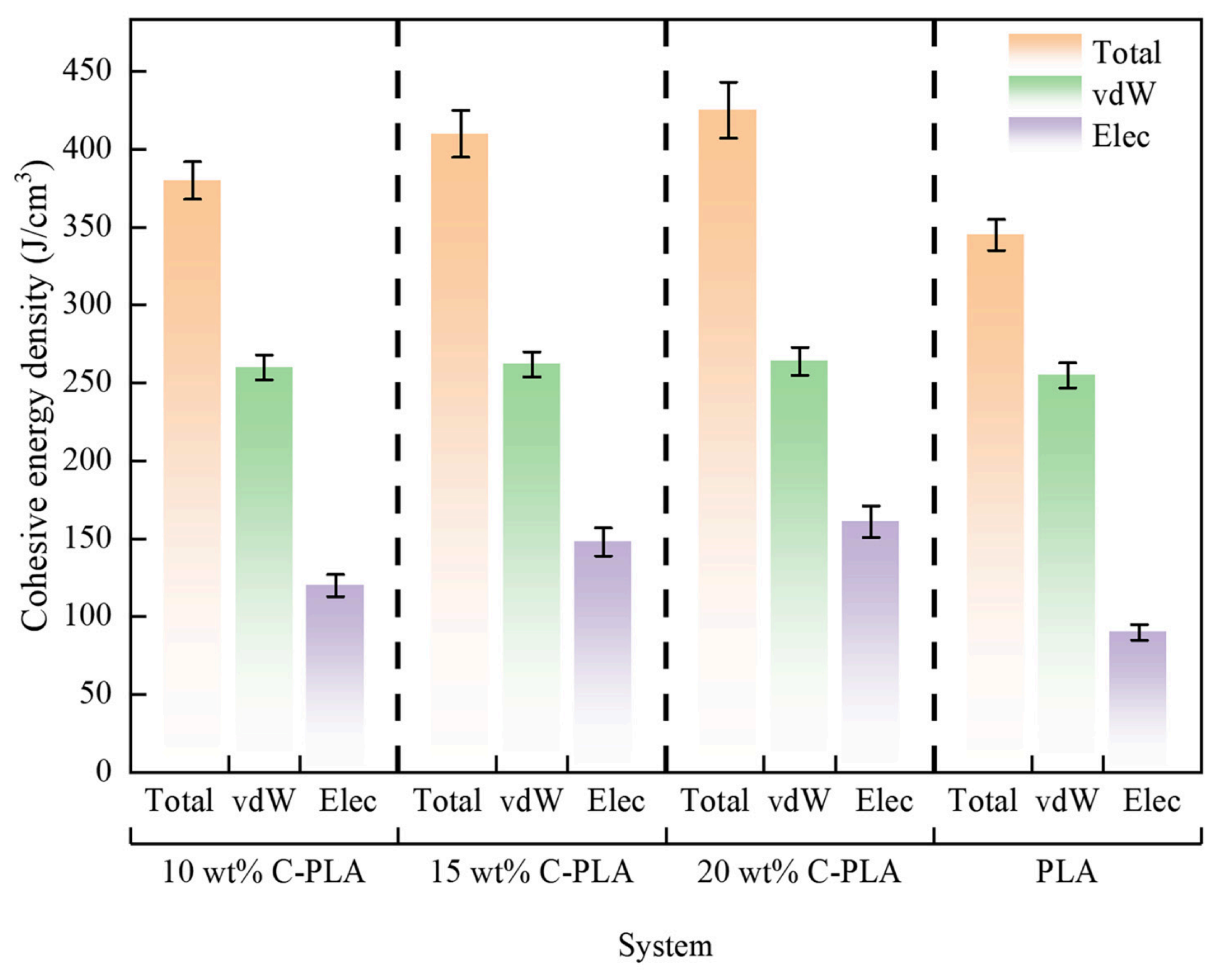
CED and its vdW and electrostatic components for C-PLA with different cellulose loadings.

Within polymer molecular chains, intermolecular forces are essentially a comprehensive manifestation of various interactions. Owing to the extremely high molecular weight and inherent polydispersity of polymers, the interactions between macromolecular chains cannot be adequately characterized by a single force parameter. Instead, the cohesive energy or cohesive energy density (CED) is commonly employed to quantify the strength of intermolecular interactions between macromolecules. Specifically, CED is defined as the energy required to transfer one mole of molecules from the condensed state to the gas phase, divided by the molar volume, i.e., CED = ΔE_V_/V, where ΔE_V_ is the molar energy of vaporization and V is the molar volume. The square root of CED gives the Hildebrand solubility parameter, δ = CED^1^/^2^. All CED values presented in Figure 7 were calculated using the Forcite module of Materials Studio via the direct nonbonded energy analysis method. In this approach, CED is obtained as the sum of the vdW and electrostatic non-bonded energies of the entire simulation cell divided by the cell volume, i.e., CED = (E_vdW_ + E_ele_)/V. Single-point energy calculations were performed on the equilibrated amorphous cells under the NVT ensemble with periodic boundary conditions following the standard Forcite protocol.

As shown in Figure 7, the total CED of the pure PLA system is 345 J/cm^3^, with the electrostatic contribution accounting for only 26%, indicating that intermolecular interactions in the pure PLA matrix are dominated by vdW forces. The total CED increases monotonically with cellulose content: 380 J/cm^3^ for the 10 wt% system, 410 J/cm^3^ for the 15 wt% system, and 425 J/cm^3^ for the 20 wt% system. In striking contrast to the nearly constant vdW contribution (255–264 J/cm^3^), the electrostatic component increases significantly with cellulose loading: from 120 J/cm^3^ at 10 wt% to 148 J/cm^3^ at 15 wt% (a 23% increase), and further to 161 J/cm^3^ at 20 wt%. The corresponding standard deviations (as represented by the error bars in Figure 7) are sufficiently small relative to these variations, confirming that the observed differences are statistically significant. This trend clearly demonstrates that the increase in the total CED of the composite systems originates almost exclusively from the enhancement of electrostatic interactions.

Notably, the electrostatic CED of the 15 wt% system (148 J/cm^3^) exhibits a 23% leap relative to that of the 10 wt% system (120 J/cm^3^), with the electrostatic fraction increasing from 32% to 36%. This corresponds to the maximum peak height of $g_{H\text{-}O}^{\mathrm{inter}}\left(r\right)$ and the highest hydrogen bond density per unit volume identified in the RDF analysis, indicating that the continuous network formed at 15 wt% cellulose maximizes the interfacial hydrogen bond density and achieves peak efficiency in electrostatic interactions. Although the electrostatic CED of the 20 wt% system continues to increase to 161 J/cm^3^, the growth rate slows considerably (the electrostatic fraction rises only marginally to 38%), which can be attributed to cellulose self-aggregation weakening the effective electrostatic contact with PLA. This trend is consistent with the findings reported by Ren et al. [34].

### 3.2. Sintering Kinetics Analysis

#### 3.2.1. Radius of Gyration and Conformational Evolution of Molecular Chains

**Figure 8:**
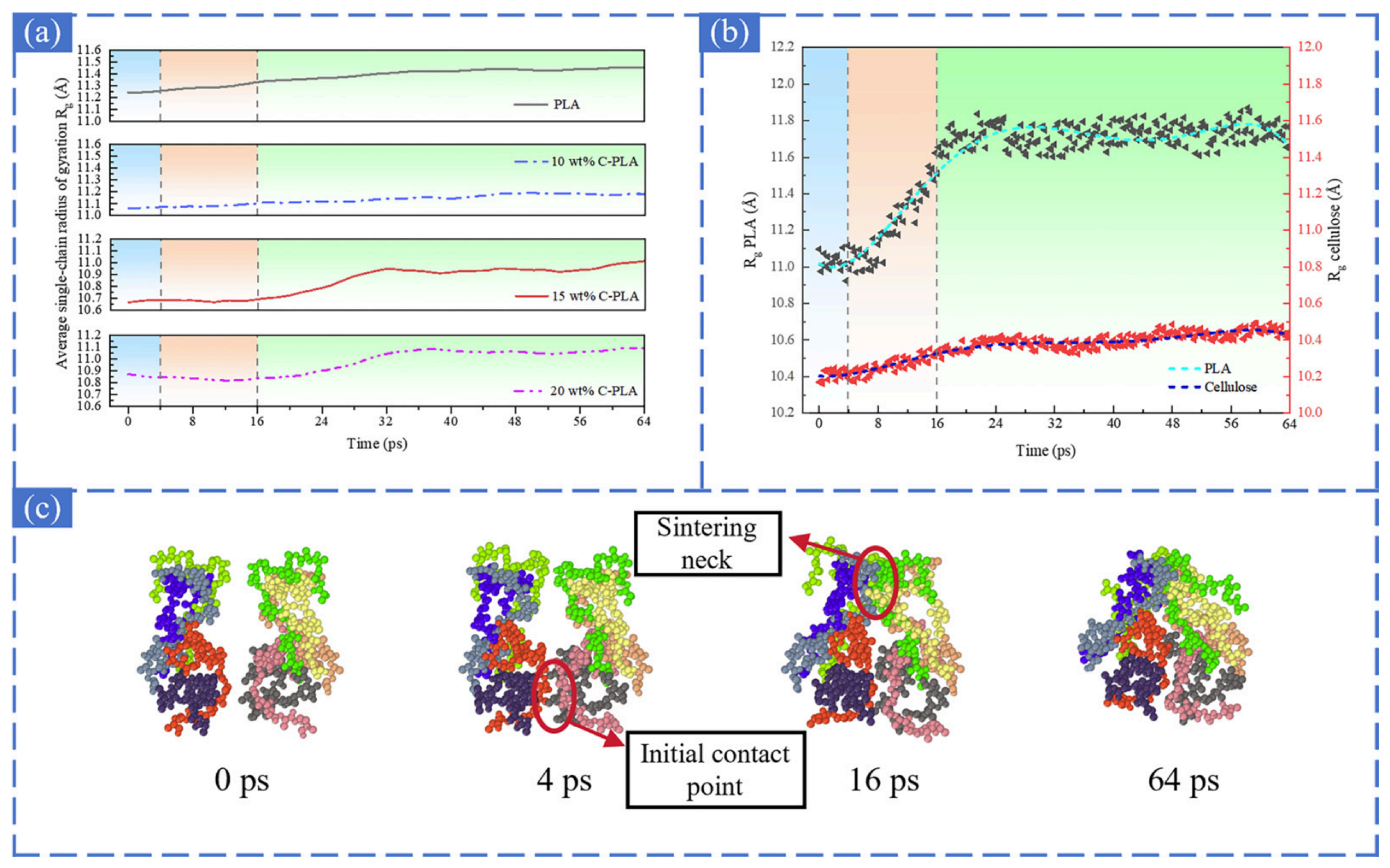
Conformational evolution analysis of polymer chains during two-nanoparticle sintering: (**a**) Evolution of average Rg for all systems; (**b**) Comparison of Rg evolution for PLA and cellulose chains in the 15 wt% C-PLA system; (**c**) Molecular conformation snapshots of the 15 wt% C-PLA system at different sintering stages.

The radius of gyration (R_g_) is a key conformational parameter that characterizes the spatial extension of polymer molecular chains, defined as the root-mean-square distance of all atoms from the center of mass of the chain. All R_g_ values were calculated using the LAMMPS compute gyration command with per-chain averaging. During the sintering of nanoparticles, the evolution of R_g_ reflects the stretching of molecular chains from a coiled conformation and serves as an effective indicator of the degree of interfacial fusion and interchain entanglement density. As shown in Figure 8a, consistent with the evolution of system potential energy and temperature, the entire sintering process can be divided into three characteristic stages. During Stage I, only vdW-driven mutual approach of the nanoparticles occurs, and the R_g_ values of all systems remain essentially constant. In Stage II, the temperature is linearly increased to 550 K, providing the polymer segments with sufficient thermal energy to overcome local rotational energy barriers; consequently, chain mobility is significantly enhanced, leading to a rapid increase in R_g_ that corresponds to the sintering neck formation period. Upon entering Stage III of isothermal sintering, R_g_ gradually approaches saturation, indicating that long-range chain diffusion progressively slows down and interfacial densification tends to stabilize. The final R_g_ increments vary considerably among the systems: the 15 wt% C-PLA system exhibits the largest increase (approximately 8.1%), whereas the increase for the 20 wt% C-PLA system (approximately 5.0%) is even lower than that of pure PLA (approximately 5.2%). This result demonstrates that cellulose content exerts a dual regulatory effect on the conformational stretching of PLA chains: an appropriate amount of cellulose (15%) provides a dense and continuous interfacial hydrogen-bond network that guides PLA chains to extend directionally along anchoring points, i.e., the cellulose acts as a rigid backbone that effectively promotes the oriented alignment of the matrix molecular chains.

**Figure 9:**
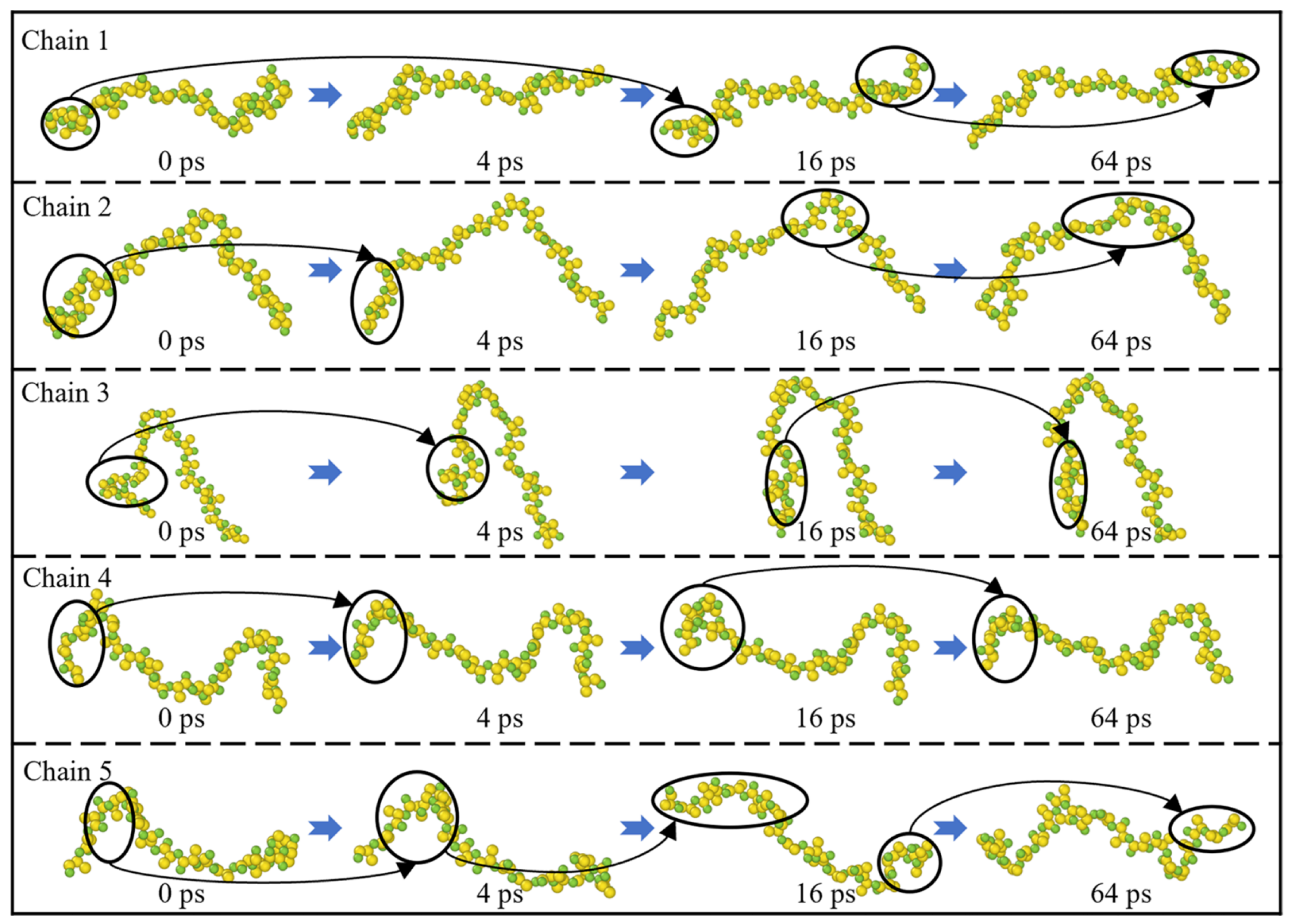
Conformational changes in chains C1–C5 during the sintering process.

Figure 8b further dissects the independent R_g_ evolution trajectories of PLA and cellulose molecular chains for the representative 15 wt% C-PLA system. The R_g_ of PLA chains displays an S-shaped growth profile similar to that of the overall system, with a final-state increase of approximately 10.1%. In stark contrast, the R_g_ of the cellulose chains remains nearly horizontal, exhibiting an absolute variation of merely 0.2 Å before and after sintering and a fluctuation amplitude of less than 2%. For reference, the standard deviation of the R_g_ measurement for PLA chains under the same equilibrated conditions is approximately 0.25 Å, indicating that the observed R_g_ variation in cellulose chains falls within the expected noise floor and confirms their rigid behavior. This difference originates from the unique molecular architecture of cellulose: its rigid pyranose ring backbone and abundant intra- and intermolecular hydrogen-bond network impart extremely high chain stiffness, enabling it to maintain conformational stability under thermally activated conditions. This observation directly proves that cellulose serves as a “rigid anchor” during sintering; it does not participate in conformational rearrangement or diffusion, but instead imposes topological constraints on PLA segments through interfacial hydrogen bonds, thereby achieving enhanced local chain stretching. The four conformational snapshots in Figure 8c and the independent molecular chain conformational changes in Figure 9 visually corroborate the above quantitative analysis. At the early stage of sintering (0 ps), the molecular chains adopt a tightly coiled globular conformation. As sintering proceeds to 16 ps, under the combined effects of interfacial hydrogen bonds and thermal activation, the molecular chains gradually extend along the normal direction of the interface, and chain ends begin to cross the sintering neck and interpenetrate into the adjacent particle. By the final sintering state at 64 ps, the molecular chains have become fully extended, spanning the original particle boundaries and forming interpenetrating entanglements with neighboring particles.

**Figure 10:**
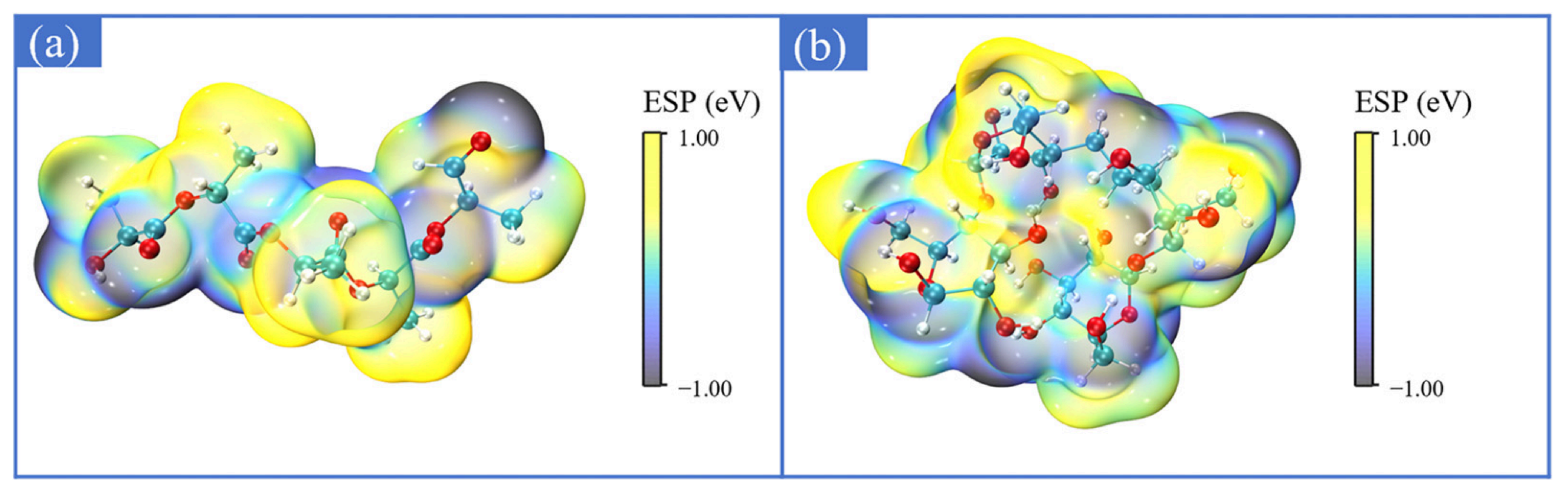
ESP surfaces: (**a**) PLA molecular chain; (**b**) cellulose molecular chain.

Meanwhile, the electrostatic potential (ESP) distribution at the molecular chain ends, shown in Figure 10 further reveals the microscopic driving force for the chain stretching process described above: the carboxyl groups at the PLA chain ends show a positive electrostatic potential (yellow regions), whereas the cellulose hydroxyl groups exhibit a negative electrostatic potential (blue regions). The strong electrostatic complementarity between them causes the terminal segments to be driven by the interfacial electrostatic potential gradient, preferentially unfolding and diffusing toward the interfacial region.

#### 3.2.2. Atomic Displacement and Migration Analysis

**Figure 11:**
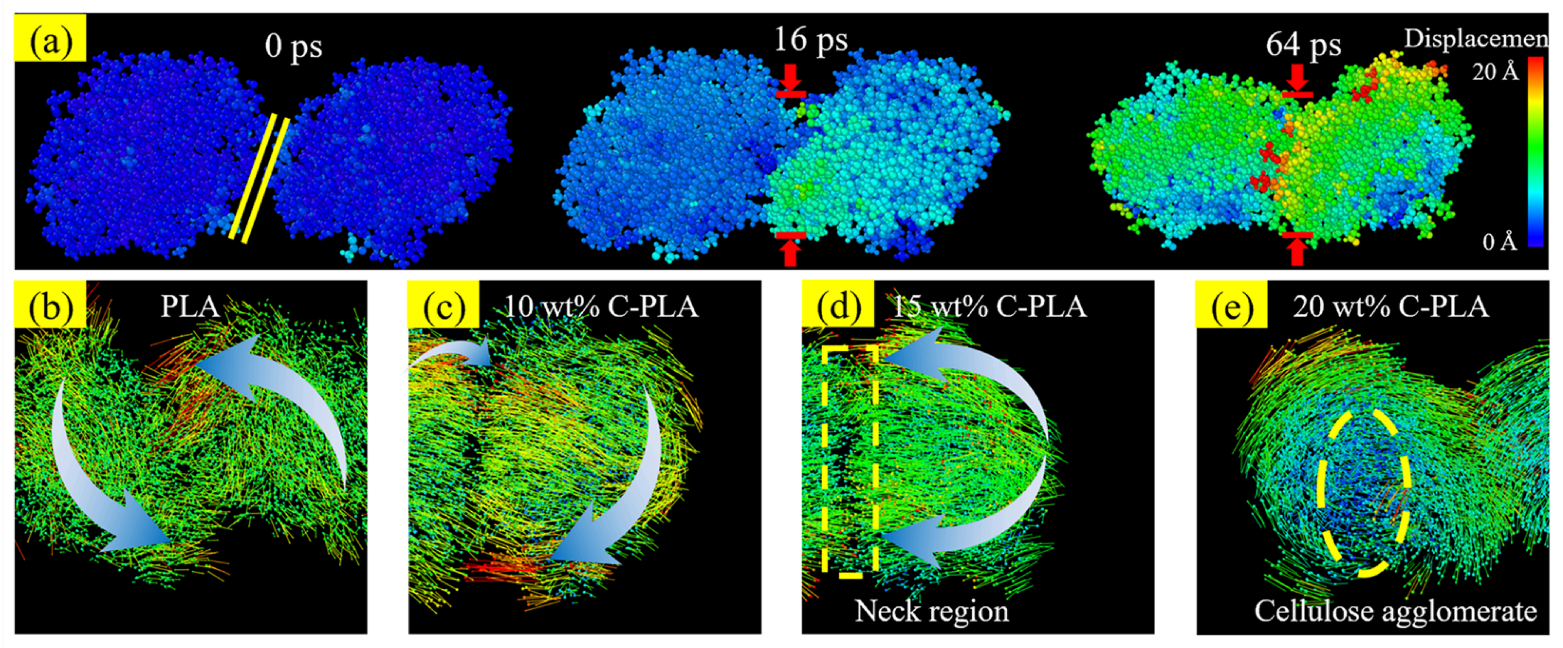
Atomic migration behavior of cellulose/PLA composite nanoparticles during sintering. (**a**) Atomic displacement maps of the 15 wt% C-PLA system at 0 ps, 16 ps, and 64 ps; (**b**–**e**) Atomic displacement vector maps of the PLA, 10 wt% C-PLA, 15 wt% C-PLA, and 20 wt% C-PLA systems at 64 ps of sintering.

To gain deeper insight into the formation mechanism of the sintering neck, Figure 11 presents the atomic displacement maps and migration vector maps of the nanoparticles. Visualization analysis of atomic displacements and migration vectors was performed using OVITO. When the system reached equilibrium at 300 K (Figure 11a), the atomic displacements were nearly zero. Upon heating to 16 ps, a sintering neck emerged, indicating the onset of thermally activated segmental migration, accompanied by continuous rotation and mutual approach of the nanoparticles. By the end of isothermal sintering at 64 ps, the high-displacement region had continuously stretched along the interface normal and covered the entire sintering neck, exhibiting a continuous gradient distribution, and the sintering interface had fully coalesced. From the migration vector maps, it can be observed that the system displays a continuous gradient with the sintering neck as the high-displacement region; the red regions are concentrated at the center of the sintering neck and gradually transition to green toward the interiors of both particles in a smooth and symmetric manner.

After the introduction of 10 wt% cellulose (Figure 11c), the displacement distribution remained largely continuous; however, the high-displacement red region shrank slightly and a few blue spots appeared inside the particles, indicating that the addition of cellulose mildly perturbed the homogeneous migration of PLA chains without disrupting the displacement continuity. The 15 wt% C-PLA system (Figure 11d) exhibited the most desirable displacement characteristics—the red high-displacement region in the sintering neck reached its maximum in both width and depth, and the displacement decayed smoothly in a stretched manner from the sintering neck toward the particle interiors, with a continuous and uninterrupted color transition. This indicates that the cellulose network guided the directionally enhanced migration of PLA chains along the interface normal through interfacial hydrogen-bond anchoring sites. This phenomenon is consistent with the “filler network effect” theory proposed by Jouault et al. for polymer-filler systems—when active fillers form a percolating network within the polymer matrix, they can synergistically enhance the mobility of local chain segments through interfacial interactions [35]. In contrast, the 20 wt% C-PLA system (Figure 11e) exhibited a pronounced “isolated island” phenomenon in its displacement distribution: numerous isolated blue low-displacement patches (displacement ≤ 2 Å) were scattered around the sintering neck and within the particles, fragmenting the originally continuous high-displacement region (red) into discontinuous patches. The displacement gradient interfaces displayed abrupt changes rather than smooth transitions, confirming the inhibitory effect of cellulose self-aggregation on the long-range interpenetration of PLA chain segments.

## 4. Conclusions

This study elucidates the interfacial interaction mechanisms and sintering kinetics of cotton stalk cellulose-reinforced PLA composites during SLS through all-atom molecular dynamics simulations. The interfacial analysis reveals that hydrogen bonds formed between cellulose hydroxyl groups and PLA carbonyl groups serve as the primary driving force for interfacial binding. With increasing cellulose content, the interfacial hydrogen bond density exhibits a non-monotonic trend of an initial enhancement followed by a decline, as evidenced by the cross-RDF peak intensity rising from 2.18 at 10 wt% to a maximum of 3.85 at 15 wt% before dropping to 1.78 at 20 wt%. A moderate amount of cellulose can establish a continuous and dense interfacial hydrogen-bond network while suppressing unfavorable dipole–dipole stacking between PLA chains, whereas excessive cellulose induces self-aggregation that weakens heterogeneous interfacial contact. This compositional dependence is further quantified by the electrostatic contribution to CED, which exhibits a 23% increase from 10 wt% to 15 wt%, consistent with the optimized hydrogen-bond network at the latter composition. Consequently, an optimal cellulose composition exists that maximizes the interfacial electrostatic interactions.

The sintering kinetics analysis demonstrates that the nanoparticle coalescence process can be divided into three characteristic stages: in the initial stage, vdW forces drive the mutual approach of the particles; in the intermediate stage, chain mobility is significantly enhanced with increasing temperature, leading to the formation and growth of the sintering neck; and in the final stage, interfacial densification is achieved through long-range chain diffusion and entanglement. Throughout the sintering process, the conformation of cellulose molecular chains remains stable, and they function as rigid anchors that guide PLA chains to extend directionally along the interface normal via interfacial hydrogen bonds. The system with the optimal composition exhibits a continuous and uniform atomic migration distribution in the sintering neck region, whereas the system with excessive cellulose displays impeded migration due to self-aggregation, accompanied by the appearance of isolated low-displacement regions.

It should be noted that the PLA chains used in this study (DP = 20, M_w_ ≈ 1440 g/mol) are significantly shorter than those in commercial PLA grades (e.g., 4032D, M_w_ ≈ 100,000 g/mol), and thus chain entanglement effects—which are important in real PLA melt sintering—are largely absent in the present model. Nevertheless, the observed trends in interfacial hydrogen-bonding and composition-dependent chain mobility are expected to be qualitatively transferable, as these interactions operate at the local segmental level and are less sensitive to overall chain length.

Beyond composition design, this work provides a mechanistic framework that links interfacial hydrogen-bonding, chain conformational dynamics, and atomic migration to the macroscopic sintering outcome. This framework is generalizable to other plant-fiber/polymer systems processed by SLS, offering a predictive basis for formulating bio-based composite powders. More broadly, the methodology demonstrated here—combining force-field-based molecular dynamics with multi-scale analysis of interfacial and kinetic parameters—can be extended to guide the development of other thermally processed polymer composites where interfacial interactions dictate performance. Future work could further explore the influence of fiber aspect ratio, surface modification, and processing parameters on the sintering behavior, building upon the mechanistic foundation established in this study.

## Figures and Tables

**Table 1:** Details of the two-nanoparticle models for sintering simulations.

System	DP		Number of Chains		Molar Mass (g/mol)		Total Molar Mass (g/mol)	Molar Mass (g/mol)	
	PLA	Cellulose	PLA	Cellulose	PLA	Cellulose		PLA	Cellulose
PLA	20	0	19	0	27,460	0	27,460	100.00	0.00
10 wt% C-PLA	20	4	17	2	24,569	2725	27,294	90.02	9.98
15 wt% C-PLA	20	4	16	3	23,124	4088	27,212	84.98	15.02
20 wt% C-PLA	20	4	15	4	21,679	5450	27,129	79.90	20.10

## Data Availability

The original contributions pre-sented in this study are included in the article. Further inquiries can be directed to the cor-responding authors.

## Abbreviations

The following abbreviations are used in this manuscript:

C-PLA	Plant fiber/PLA composites
CED	Cohesive energy density
DP	degree of polymerization
ESP	Electrostatic potential
PBS	Polybutylene succinate
PEG	Poly(ethylene glycol)
PLA	Polylactic acid
RDF	Radial distribution function
Rg	Radius of gyration
SLS	Selective laser sintering
vdW	van der Waals
